# Monogenic susceptibility to live viral vaccines

**DOI:** 10.1016/j.coi.2021.05.006

**Published:** 2021-10

**Authors:** Florian Gothe, Sophie Howarth, Christopher JA Duncan, Sophie Hambleton

**Affiliations:** 1 Immunity and Inflammation Theme, Translational and Clinical Research Institute, Newcastle University, UK; 2 Department of Pediatrics, Dr. Von Hauner Children’s Hospital, University Hospital, Ludwig-Maximilians-Universität Munich, Munich, Germany; 3 Infection and Tropical Medicine, Royal Victoria Infirmary, The Newcastle upon Tyne Hospitals NHS Foundation Trust, UK; 4 Children’s Immunology Service, Great North Children’s Hospital, The Newcastle upon Tyne Hospitals NHS Foundation Trust, UK

## Abstract

Live attenuated viral vaccines (LAV) have saved millions of lives globally through their capacity to elicit strong, cross-reactive and enduring adaptive immune responses. However, LAV can also act as a Trojan horse to reveal inborn errors of immunity, thereby highlighting important protective elements of the healthy antiviral immune response. In the following article, we draw out these lessons by reviewing the spectrum of LAV-associated disease reported in a variety of inborn errors of immunity. We note the contrast between adaptive disorders, which predispose to both LAV and their wild type counterparts, and defects of innate immunity in which parenterally delivered LAV behave in a particularly threatening manner. Recognition of the underlying pathomechanisms can inform our approach to disease management and vaccination in a wider group of individuals, including those receiving immunomodulators that impact the relevant pathways.

**Current Opinion in Immunology** 2021, **72**:167–175

This review comes from a themed issue on **Host pathogen**

Edited by **Helen C Su** and **Jean-Laurent Casanova**

For a complete overview see the Issue and the Editorial

Available online 6th June 2021

**https://doi.org/10.1016/j.coi.2021.05.006**

0952-7915/© 2021 The Authors. Published by Elsevier Ltd. This is an open access article under the CC BY license (http://creativecommons.org/licenses/by/4.0/).

The advent of COVID-19 has highlighted the importance of improving our working knowledge of antiviral immunity in humans, in order to predict, manage and protect against severe viral disease. Widespread implementation of live viral vaccination has been exceptionally effective as a public health intervention against previously endemic infectious diseases such as smallpox, measles and polio. Live attenuated viral vaccines (LAV), showing reduced pathogenicity and replication compared with the parent virus in healthy hosts, retain molecular patterns that agonise innate inflammatory pathways, providing an inbuilt ‘adjuvant’ to boost the immunogenicity of viral antigens. By comparison with inactivated or subunit vaccines, LAV typically elicit broader and more durable immune responses, resulting in superior protection of individuals and populations. Yet it has long been recognised that extremely rare inborn errors of immunity (IEI) enable attenuated viruses to behave as pathogens in affected individuals. As a result, immunodeficiency is a contraindication to receipt of LAV and associated dissemination is almost by definition a sentinel event. Dissecting the molecular pathogenesis of these extreme infection phenotypes continues to shed light on the non-redundant roles of distinct elements of the antiviral immune response, and may inform understanding of viral immune evasion.

LAV currently in widespread use derive from a wide range of pathogens, from non-enveloped RNA viruses (such as Rotavirus, RoV) all the way to a complex enveloped DNA virus that establishes latency within even immunocompetent hosts (Varicella zoster virus, VZV) (Table 1). Reflecting this diversity, perhaps it is no surprise that susceptibility to disease caused by LAV (Figure 1) shows fine specificity among immunodeficiency states including combined immunodeficiency, predominantly antibody deficiencies and disorders of innate antiviral immunity (Table 2). We now discuss each LAV in turn, omitting those for which IEI-associated viral dissemination has not been reported, presumably because of historic or restricted use (Vaccinia; Adenovirus) or extreme attenuation (Influenza).

**Table 1:** Characteristics of live-attenuated viral vaccines in current or recent clinical use

Virus	Year licensed	Method of attenuation	Route	Schedule	Combined preparations	Strain(s)	Products (examples)
Influenza	2003	Reassortants of cold-adapted (ca) temperature sensitive (ts), attenuated (att) MDV and wt *H* and *N* segments	Nasal	1−2 dose	–	Reassortants of IAV (H1N1, H3N2) and two IBV strains	FluMist Quadrivalent Fluenz Tetra
Measles	1963	*In vitro* passage	SC/IM	2 dose	MR	Enders’ attenuated Edmonston B	Attenuvax
					MMR	Schwartz	
					MMRV	Edmonston-Zagreb	MMR:
							MMRVaxPro, Priorix, MMR-II + others
Mumps	1967	*In vitro* passage	SC/IM	2 dose	MMR	Jeryl-Lynn^TM^ (B level)	Mumpsvax
					MMRV	Jeryl-Lynn^TM^ RIT 4385	
						Urabe AM9	See MMR/MMRV
Polio	1961−63	*In vitro* passage	Oral	2−3 dose	–	Sabin strains:	Trivalent OPV (tOPV)
						type 1 (LS-c, 2ab)	Bivalent OPV (bOPV)
						type 2 (P712, Ch, 2ab)	
						type 3 (Leon 12ab)	
Rotavirus	2006^a^	*In vitro* passage	Oral	2 dose	–	Rotarix: G1P[8] RIX4414 strain	Rotarix, RotaTeq + others
		(Rotarix)				RotaTeq: G1, G2, G3, G4, P1A[8]	
		Human-bovine reassortants (RotaTeq)					
Rubella	1969	*In vitro* passage	SC/IM	1−2 dose	MR	Wistar RA 27/3	See MMR/MMRV
					MMR		
					MMRV		
Varicella	1984	*In vitro* passage	SC	Varicella: 1−2 dose	MMRV	Oka	Varicella:
							Varivax, Varilix
				Zoster: 1 dose			Zoster:
							Zostavax
							MMRV:
							ProQuad, PriorixTetra
Yellow Fever	1938	*In vitro* passage	IM/SC	1 dose	–	17D (derived from Asibi strain)	YF-Vax, Stamaril

MDV = master donor virus - generated by laboratory adaptation and/or reverse genetics.

MMR - co-formulation of measles, mumps and rubella (MMR) - approved in 1971, largely replacing individual vaccines.

MMRV - co-formulation of measles, mumps, rubella and varicella (MMRV) - approved in 2005.

a The Rotashield vaccine was licensed in 1998 then withdrawn in 1999 due to concerns regarding intussusception.

**Figure 1:**
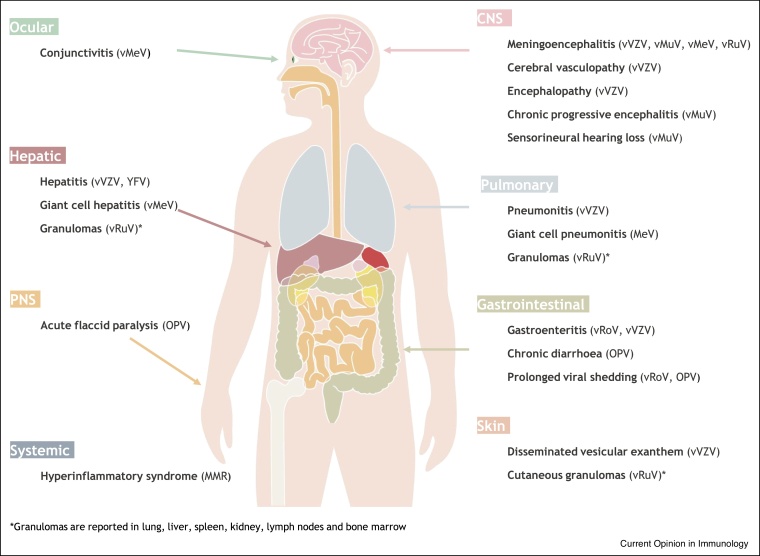
Disease caused by live attenuated vaccines in children with primary immunodeficiency.

**Table 2:** Pattern of susceptibility to live attenuated vaccines according to IUIS category and subcategory of IEI

IUIS disease category	Inborn error of immunity	Live viral vaccine							References
		Measles	Mumps	Rubella	Varicella	Rotavirus	Yellow fever	Poliovirus	
1. Immunodeficiencies affecting cellular and humoral immunity	T-B+ SCID			x	x	x			[5−9,46]
	T-B- SCID		x	x	x	x		x	[5,9,41,50,51,56,62,85]
	CID			x				x	49, 50, 51,84]
2. Combined immunodeficiencies with associated or syndromic features	DNA repair defects			x					[48, 49, 50, 51,52^•^]
	DiGeorge syndrome			x					[52^•^]
	Cartilage hair hypoplasia			x					[49,51]
3. Predominantly antibody deficiencies	Agammaglobulinemia							x	[76,86, 87, 88]
	APDS^a^			x	x				[48,50,58,59]
6. Defects in intrinsic and innate immunity	WHIM			x					[51]
	Predisposition to severe viral infection	x	x	x	x		x		[26,27^••^,^29^,^39^,^40^,^67^,72^•^]

a Arguably, APDS is more correctly classified as a combined immunodeficiency with syndromic features.

## Rotavirus

Rotaviruses (RoV) were discovered as a cause of acute gastroenteritis in infants in 1973 [1]. They are non-enveloped, double-stranded RNA viruses belonging to the family of *Reoviridae* [2]. RoVs primarily infect mature enterocytes of the small intestine, leading to malabsorption and diarrhoea [2,3]. Although more than 100 countries worldwide now vaccinate against them, RoV infections still account for >120 000 deaths per year in pre-school children, mostly in low-income countries [4].

Current vaccination strategies use orally administered LAV such as Rotarix and RotaTeq. Over the years, severe or prolonged vaccine strain RoV (vRoV) infections have been reported in several patients with severe combined immunodeficiencies (SCID), both T-B- as well as T-B+ forms [5, 6, 7, 8, 9] (Table 2). In line with the notion that T cell immunity is essential to RoV-clearance, low levels of RoV-specific CD8+ T cells can be detected in immunocompetent children with acute RoV disease [10]. Although horizontal transmission of vRoV has been detected, it was not associated with gastrointestinal symptoms [11]. Chronic diarrhoea caused by wild-type RoV has also been reported in SCID patients [12, 13, 14], and also in other combined immunodeficiencies (CID) like Di George syndrome [14,15] and Cartilage hair hypoplasia [15,16] as well as in one patient with X-linked agammaglobulinaemia (XLA) [12].

Since newborn screening for SCID is only available to a small minority of children today, the parenteral non-replicating RoV vaccine currently evaluated in a phase III trial (NCT04010448) might reduce the risk of severe infection in undiagnosed SCID patients [17^•^]. However, recent data from Australia [18^••^] and the US [19] reported a reduced incidence of type I diabetes since the introduction of oral RoV vaccination programs, potentially representing a second important rationale for retention of the LAV.

## Measles

Measles virus (MeV), a single-stranded negative sense RNA virus, causes a self-limiting illness with a characteristic erythematous maculopapular rash appearing first on the face and behind the ears. Before the introduction of a highly effective LAV, MeV was associated with up to 50% percent of infectious disease-related childhood deaths, albeit most were not caused by MeV directly [20], but by bacterial superinfection. This secondary susceptibility, commonly resulting in otitis media or pneumonia, would sometimes manifest years after measles and correlate with a contraction in the humoral immune repertoire not seen in children receiving the MMR vaccine [20,21,22^••^,^23^] Nonetheless, natural immunity after measles is classically lifelong. As a neurotropic virus, wild-type MeV is associated with serious central nervous system (CNS) disease: post-MeV encephalomyelitis occurs in about 0.1% of patients within weeks after primary infection [24]. Months to years after exposure, subacute sclerosing panencephalitis (SSPE) occurs in approximately 1 in 10 000–100 000 cases, reflecting persistent CNS infection [24]. Measles inclusion body encephalitis is similarly rare and is associated with infection of immunocompromised hosts.

Originally developed as a single vaccine, MeV vaccination is now provided in combination with mumps and rubella (as MMR), sometimes with the addition of varicella vaccine (as MMRV). Despite the administration of hundreds of millions of doses of MMR worldwide every year, pathological dissemination of vaccine strain MeV (vMeV), or indeed mumps or rubella, is an extremely unusual occurrence and acts as a ‘red flag’ for underlying IEI. This is particularly true of defects of type I and type III interferon (IFN-I and IFN-III) immunity, where disease due to vMeV may be the presenting feature. End-organ disease associated with vMeV includes pneumonitis and/or hepatitis, as well as encephalitis. Pneumonitis and hepatitis were observed in three individuals with homozygous STAT2 deficiency — which abrogates signalling in response to both IFN-I and IFN-III [25,26]. Encephalitis post-MMR, associated with detection of vMeV in CSF, has been reported in homozygous IFNAR1 [27^••^] deficiency. Both IFNAR1 and IFNAR2 are essential for signalling in response to all IFN-I subtypes, reflecting the relevance of IFN-I to protection of the CNS. Life-threatening illness including encephalitis, in temporal association with MMR administration, has also been reported in homozygous deficiency of STAT1, STAT2 and IFNAR1, although vaccine origin could not be definitely proven [28, 29, 30,31^••^]. In addition, an emerging phenotype of hyperinflammation, sometimes meeting diagnostic criteria for haemophagocytic lymphohistiocytosis (HLH), is associated with MMR vaccination in patients with homozygous defects in IFNAR1, IFNAR2, STAT1 and STAT2 [28,30,32,33]. Pathological dissemination of vMeV is less commonly the presenting feature of significant defects of T cell immunity (e.g. SCID or CID), possibly because these diseases typically manifest before the administration of MMR, at 12–15 months of age. Nevertheless, both pneumonitis and hepatitis have been observed in vaccinated SCID and CID patients, underscoring the added importance of T cell immunity to vMeV host defense [34, 35, 36, 37].

## Mumps

Painful swelling of the parotid gland, usually starting unilaterally, is considered the hallmark of mumps [38]. Although generally causing a self-limiting illness, MuV is neurotropic and therefore neurological manifestations such as aseptic meningitis (1–10%), encephalitis (0.1%) and sensorineural hearing loss are well recognised [38]. Proven vaccine strain MuV (vMuV) disease is extremely rare and, like vMeV, is mainly seen in inborn errors of IFN-I/III immunity. In a case of homozygous IFNAR2 deficiency with fatal encephalitis after MMR, vMuV RNA was detected (alongside vaccine-strain rubella) in molecular analysis of brain tissue [39]. In homozygous STAT2 deficiency, vMuV has also been detected in CSF of a patient with meningoencephalitis post-MMR [40]. Sensorineural hearing loss, potentially suggestive of vMuV involvement, was reported in an additional STAT2-deficient child post-MMR [25]. Chronic progressive encephalitis, with detection of vMuV in the brain, was also seen in a child with RAG1 deficiency following HSCT [41]. Thus, both IFN-I/III and T cell immunity appear important for control of vMuV, as for vMeV. This is true also of rubella virus (vRuV).

## Rubella

RuV is a single-stranded negative-sense RNA virus and part of the newly formed family of *Matonaviridae* [42]. Postnatal infections are transmitted via inhalation of aerosols with RuV replication taking place in cells of the upper respiratory tract [43]. Most cases show a mild, self-limiting disease course, however, in women with primary RuV infection during the first trimester of pregnancy miscarriage or congenital rubella syndrome (CRS) are frequently observed [43,44]. In addition to the well-known CRS manifestations, some infants develop a multisystem-inflammatory disease with chronic rubella-like rash, persistent diarrhoea and pneumonitis within the first year of life [45,46]. In IEI patients, infections with wild-type RuV have, like MeV and MuV, not been reported to cause relevant illness, presumably due to the rarity of wild type infection in the population — reflecting the success of vaccination campaigns.

The RuV vaccine was developed by serial passaging of a patient-derived strain through human diploid cell lines [47] and is usually given as part of the MMR or MMRV vaccine. In 2014, the first report of vaccine-strain RuV (vRuV) detection in cutaneous granulomas of IEI patients was published by Bodemer *et al.* [48]. Subsequent studies localized vRuV within M2 macrophages at the center of granulomas [49], which have also been observed in visceral organs including lung, spleen, kidney, lymph nodes, bone marrow, and liver [50,51]. Affected patients mostly suffered from profound T cell immunodeficiency associated with DNA repair disorders [48, 49, 50, 51,52^•^,^53^] or SCID [50,51,54] (Table 2). Additionally, patients with vRuV+ granulomas and underlying Cartilage hair hypoplasia [49,51], WHIM syndrome [51], MHC class II deficiency [51], APDS [48,50] and further combined immunodeficiencies without molecular diagnosis [49, 50, 51] have been published (recently reviewed in Ref. [52^•^]). Inflammatory granulomas appeared 2–152 months post vaccination indicating vRuV persistence for years [51]. By contrast, widespread dissemination of vRuV, including to the CNS, occurred within three weeks of MMR vaccination in a child with fatal encephalitis and IFNAR2 deficiency [39]. This information, albeit preliminary, suggests that IFN-I may be important in initial control of vRuV (particularly in the CNS), but that T cell responses are also essential for resolving and/or controlling persistent infection.

## Varicella zoster virus

Varicella zoster virus (VZV) is an alpha-herpesvirus that causes distinct exanthematous syndromes upon primary infection (chickenpox, varicella) or reactivation from latency (shingles, zoster). Although typically a mild and self-limiting febrile illness, chickenpox can cause a range of life-threatening complications including dissemination to the lungs, liver and brain, especially in the immunocompromised. These considerations, and the huge societal costs imposed by a universal contagious childhood illness, provided the rationale for development of a live attenuated VZV vaccine by serial passage of a disease isolate in tissue culture (‘Oka strain’, vVZV). Its incorporation into the universal childhood vaccination schedule in the US in 1995 transformed the epidemiology of VZV in that population and has since been adopted by many countries. Two doses of vVZV, generally given alongside or in combination with MMR, are approximately 95% effective in preventing varicella in healthy children. The same Oka strain, administered in a higher dose to older individuals, boosts cellular immunity to VZV and reduces rates of zoster, especially complicated disease. Much lower circulation of VZV lends herd immunity against both varicella and zoster to unvaccinated immunocompromised individuals.

Although attenuated, vVZV replicates in the host and causes local skin vesicles in a small minority of otherwise healthy recipients, but is nonetheless extremely safe relative to wild type varicella [55]. Its dissemination always denotes significant immunodeficiency, whether primary (inborn errors) or secondary (such as AIDS, cancer chemotherapy etc). Disseminated vVZV is rare and more often observed in non-SCID T cell immunodeficiency states such as hypomorphic deficiencies of RAG2 [56], DOCK8 deficiency [57], APDS [58,59] or molecularly undefined CID [55,60,61] than in late-presenting true SCID [54,62,63]. A specific role for NKT cells in immunity to vVZV has been proposed based on severe disease in children with apparently selective deficiency of this innate-like T cell subset [64,65]. Severe vaccine strain varicella has also been observed in deficiency states of STAT1 [66] and STAT2 [67], consistent with a role for innate interferon in the initial restraint of vVZV replication as in mouse models [68]. Similar to the pattern of predisposition to severe wild type varicella, antibody deficiency does not in itself confer risk of vVZV dissemination.

Vaccine-strain VZV is sensitive to antiviral agents such as aciclovir and it is therefore all the more striking that fatalities have nonetheless been recorded. Very rarely, dissemination of live attenuated zoster vaccine has occurred in the immunosuppressed elderly [69]. One might predict an excess of vaccine strain zoster in vaccinated children with T cell deficiencies, similar to their increased risk of wild type zoster after varicella, but this has not so far been reported. Overall, vVZV appears less likely to reactivate to cause zoster than wild type virus, reflected in a reduced incidence of childhood zoster in highly vaccinated populations [70].

## Yellow fever

The highly effective 17D yellow fever virus (YFV) vaccine was developed some 80 years ago. Very rare life-threatening sequelae of vaccination are recognised — yellow fever vaccine-associated viscerotropic disease (YEL-AVD) or neurotropic disease (YEL-AND). These are more common in infants, the elderly, and patients with immunocompromise. Despite suspicions that YEL-AVD/YEL-AND may have a genetic component, there has been only one report of YEL-AND in an otherwise healthy adolescent with homozygous IFNAR1 deficiency [27^••^] and one report of YEL-AVD in IFNAR2 deficiency [71^•^]. Incidentally, neither individual experienced disease following receipt of MMR, suggesting that susceptibility to MMR dissemination is not fully penetrant in patients with profound defects of IFN-I immunity. Circumstantial evidence for YEL-AVD exists in another child, born to consanguineous parents carrying heterozygous deleterious IRF9 variants, who died of enterohemorrhagic fever subsequent to YFV vaccination [72^•^], the implication being that this child was IRF9 deficient. In keeping with the hypothesis that IFN-I/III immunity is important in preventing YFV vaccine-associated disease, Bastard *et al.* recently documented neutralizing autoantibodies in 3 of 8 previously healthy adults with such complications [71^•^].

## Poliovirus

The live-attenuated oral poliovirus vaccine (OPV) has driven the success of the global polio eradication initiative (GPEI) thanks to its low cost, ease of administration and ability to induce better mucosal immunity than the inactivated poliovirus vaccine (IPV) [73].

The OPV is usually given in the first few months of life. Whereas immunocompetent children clear OPV in 2–6 weeks [74], immunodeficiency may cause prolonged viral replication and excretion. Replicative nucleotide substitutions produce highly divergent immunodeficiency-associated vaccine-derived viruses (iVDPV) and potential recovery of neurovirulence. Excreted iVDPVs with high divergence in the VP1 coding region are poorly neutralised by the sera of IPV/OPV-vaccinated immunocompetent children *in vitro* [75^•^], though their potential to cause outbreaks is not yet clear [76,77^••^].

Analysis of WHO registry cases showed iVDPVs were excreted for a median duration of 1.3 years [78]. In contrast, prolonged viral excretion has not been reported in patients with HIV [79, 80, 81]. Recent data suggest CID patients, particularly those with SCID or MHC-II deficiency, have an increased propensity to prolonged iVDPV excretion [76,82]. The longest duration of excretion (>5 years), however, is reported in patients with CVID, possibly due to their milder clinical phenotype [76,78]. Corresponding with reversion to neurovirulence, acute flaccid paralysis is reported in 64% of WHO registry iVDPV cases [77^••^]. Though genetic diagnoses are not available for all reported cases, vaccine associated paralytic poliomyelitis (VAPP) has been reported in patients with both combined and humoral immunodeficiencies, including those with MHC class II deficiency [83], RAG1 deficiency [84] and XLA. A systematic review of 107 iVDPV cases found agammaglobulinaemia was associated with the highest likelihood of VAPP [76], and rates among XLA patient cohorts are reported as 0.6–3% [85, 86, 87], compared with a population frequency of 1 per 2.7 million doses [88]. Interestingly, the same group is also highly susceptible to other naturally occurring members of the enterovirus family, resulting in severe disease, particularly encephalitis. Treatment with IVIG, HSCT and the capsid inhibitor pocapavir is reported with variable success [76,77^••^,^89^].

## Conclusion

Disease caused by the continued replication of highly attenuated viral vaccines invariably denotes immunocompromise, whether inborn or acquired. Different viruses stress different parts of the antiviral response, as reflected in the spectrum of susceptibility produced by specific types of immunodeficiency. Persons with severe deficits of T cell immunity are vulnerable to disease caused by almost any LAV alongside multiple conventional and opportunistic viral pathogens. While humoral immunity appears indispensible for relatively few types of virus, enteroviruses — and hence oral polio vaccine — are among them. By contrast, the spectrum of disease in individuals with defects of innate antiviral sensing and IFN signalling indicates their importance as part of the concerted immune response to parenteral LAV including MMR, vVZV and YFV vaccines.

The degree to which the same molecular defect might predispose to severe disease caused by the parent virus can be difficult to assess owing to the success of universal vaccination programmes in reducing the circulation of wild type virus. Despite often gross impairment *in vitro*, inborn errors of innate antiviral immunity sometimes manifest clinically only upon receipt of LAV, implying retention or even possibly gain of certain virulence factors by the vaccine strain virus. One important confounding factor might be the mode of delivery: with intramuscular injection, the barrier function of mucosal immunity is bypassed, abrogating the protective role of local factors such as IFN-λ. Nonetheless, defects of IFN-I/III signalling have recently been described among previously healthy adults presenting with severe COVID-19 despite earlier exposure to LAV [90]. Furthermore, probable wild type mumps complicated by sensorineural deafness, as well as Herpes simplex encephalitis, were also described by the same group within a kindred with IFNAR1 deficiency [31^••^]. We might conclude that susceptibility to natural viral pathogens is likely increased by these innate defects but potentially mitigated by competing factors including lower exposure than to LAV. Indeed, penetrance of either aspect of viral susceptibility is incomplete.

Outstanding questions in the field of monogenic susceptibility to LAV (Box 1) thus concern aspects of host–pathogen interaction of key importance for public health. In particular, a better understanding of the role of innate immune priming might assist in the design of ‘smarter’ LAV with optimal immunogenicity, for example through elimination of viral IFN antagonists. Meanwhile, testing the behavior of LAV in a variety of IEI models could help developers improve their safety, by revealing virulence factors unmasked by immunocompromise. The complex interplay between virus and human host mandates continued alertness to extreme infection phenotypes including those associated with live vaccines.

Box 1

**Table:** 

Outstanding questions
1. Are LAVs more virulent in individuals with IEI than is their wild type parent virus?
2. What is the molecular basis of attenuation of licensed LAVs?
3. Do IEI underlie additional phenotypes of disease due to LAVs?
4. Will knowledge of the importance of IFNs in containing LAVs inform and/or improve next generation LAV development? For example, by targeted deletion of viral IFN antagonists.
5. Does parenteral, as opposed to mucosal, administration result in greater problems with dissemination of LAVs in patients with inborn errors of type I interferon immunity? Parenteral administration would potentially bypass the mucosally-restricted type III IFN response.

Alt-text: Box 1

## Funding

Related research by the authors was supported by the 10.13039/100010269Wellcome Trust (207556/Z/17/Z and 211153/Z/18/Z), the 10.13039/501100000282Sir Jules Thorn Charitable Trust (12/JTA), MRC and the Munich Clinician Scientist Program.

## Conflict of interest statement

Nothing declared.

## References and recommended reading

Papers of particular interest, published within the period of review, have been highlighted as:

• of special interest
•• of outstanding interest
